# Thai generic-brand dry canine foods: mutagenicity and the effects of feeding in vivo and in vitro

**DOI:** 10.1186/s12917-016-0640-9

**Published:** 2016-01-20

**Authors:** Tanyalak Khuntamoon, Apanchanid Thepouyporn, Sarunya Kaewprasert, Pattaneeya Prangthip, Somchai Pooudoung, Urai Chaisri, Phudit Maneesai, Karunee Kwanbunjan

**Affiliations:** Department of Tropical Nutrition and Food Science, Faculty of Tropical Medicine, Mahidol University, Bangkok, 10400 Thailand; Department of Tropical Pathology, Faculty of Tropical Medicine, Mahidol University, Bangkok, 10400 Thailand; Department of Pathology, Faculty of Veterinary Medicine, Kasetsart University, Bangkok, 10900 Thailand

**Keywords:** Dry canine foods, Mutagenicity, Long-term consumption

## Abstract

**Background:**

The commercial pet-food industry and the market value of the pet industry have increased. Most owners are concerned about their pets’ health, and prefer commercial pet foods as their regular diet. This study thus aimed to determine whether a selection of local generic-brand dry canine foods had any potential to promote chronic disease.

**Methods:**

Five local, generic-brand, dry canine foods were studied for potential mutagenicity; the effects of long-term consumption were also observed in rats. All canine foods were extracted with distilled water and absolute ethanol. The Ames test was used to detect short-term genetic damage, using *Salmonella typhimurium* tester strains TA98 and TA100. Simultaneously, the long-term effects were studied in an animal model by observing rats fed with these canine foods, compared with normal rat food, for a period of 15 weeks.

**Results:**

Using the water extracts, all dry canine foods studied showed considerable mutagenic effects on the tester strains. One brand affected both tester strains, whereas 3 showed positive to TA98, and one to TA100. With the absolute ethanol extract, three of the five brands had a considerable mutagenic effect on TA98, and another affected TA100. In the long-term test, all rats remained alive until the end of the experiment, exhibited no apparent signs of toxicity or serious illness, and maintained normal bodyweight and weight gain. Serum blood biochemistry and hematological parameters in canine food-fed rats showed some negative effects. Correspondingly, histopathological investigation of their liver and kidneys showed deterioration.

**Conclusions:**

Mutagenic potential and the negative potential health impacts were observed in all local-brand dry canine foods tested.

## Background

Dietary consumption is closely related to the development of many chronic diseases, such as obesity, cardiovascular disease, chronic renal disease, and cancer. Although the dietary mechanism related to some diseases is not clear, healthy food behaviors in aging animals can reduce the risk of many chronic diseases. Obesity is the most common form of malnutrition among dogs, estimated at 24–34 % in the United States (US) [1].

Obesity is a preventable health hazard, with health implications such as cancer, type-2 diabetes mellitus, cardiovascular complications, and decreased lifespan [2]. Most obesity cases are due to overeating and lack of exercise; this is true for both dogs and people [3]. An estimated 0.5 % of dogs in the US are diabetic; and the vast majority of these are either overweight or obese [4]. Metabolic studies from He et al. [5] indicated that obese pigs had higher serum insulin and glucagon that led to insulin resistance and dyslipidemia, similar to observations in other obese species such as rats, mice, rabbits, and human children and teenagers.

Cancer is one of the most common non-accidental causes of death for dogs and cats [6]. While the increasing prevalence of cancer among pets is multifactorial, it is also related to pets living longer. Numerous studies have outlined risk factors of certain nutrients and their relationships to the development of cancer in humans, such as decreased fiber and increased fat, which are commonly indicated as causal factors for a wide variety of malignant conditions of the gastrointestinal tract, breasts, and bladder. A study by Sonnenschein et al. [7] suggested that nutritional factors resulting in altered body composition early in life might be important in dog breast cancer.

The commercial pet-food industry is changing continuously, and the market value of the pet industry has increased from approximately 15 million US dollars in 1992 [8], to around 300 million US dollars in 2011 [9]. Most owners are concerned about their pets’ health, and prefer commercial pet foods as their regular diet. These foods are recognized as being nutritious, convenient, and of consistent quality. However, there are reasons to be concerned about the raw materials used for pet food production. For example, increased oral intake of fat, monosodium L-glutamate, and ursolic acid may introduce significant alterations in the composition of gut microbiota, which are thought to play an important role in amino acid [10], and lipid metabolism [11]. The objective of this study is to determine the safety of pet foods currently available in the market, in light of the increasing incidence of chronic diseases in pets due to their food intakes. This preliminary study could inform pet owners’ selection of appropriate diets for their pets, and the pet-food industry, potentially significantly improving the standards of companion animal health in Thailand.

## Methods

### Study design

#### Canine food

Five commonly consumed, locally manufactured, commercial dry canine foods were purchased from a market, sufficient for short-term testing and for a longer feeding experiment. For the purposes of this study, the foods were named DG diet, AP diet, AL diet, RS diet, NT diet (Table 1), and these canine foods shared the major part of the market because of their economical price. All five pet diets were extracted in crude distilled-water and absolute-ethanol and using Ames test to determine mutagenic potential.

**Table 1:** Nutritional fact of five canine foods and rat food

Nutritional facts Garanteed analysis	Control diet (Rat food)	DG diet	AP diet	AL diet	RS diet	NT diet
Protein (Min) %	24	22	27	27	26	26
Fat (Min) %	4.5	9	8	13	8	8
Ash (Max) %		10				
Calcium (Min) %	1	1	1.5		1.2	
Phosphorus (Min) %	0.9	0.8	1.25		1	
Fiber (Max) %	5	4	4	3.5	4	4
Moisture (Max) %	12	10	10	9.5	10	10
Manufacturer	S.W.T.	Petech	S.W.T.	Nutrix	Betagro	Betagro
Lot No.	EQFD082	Stn029041104	S2Ex5	8100239	1/803109	1/711071

### Mutagenicity assay

The Ames assay was used with *S. typhimurium* strains TA98 and TA100, and the well-known plate-incorporation procedure described by Maron and Ames [12]. The histidine-dependent (His-), bacterial tester strains TA98 and TA100 are capable of detecting frameshift mutation and base-pair substitute mutation, respectively. They are designed to contain a mutation in their histidine operon. Since the bacterial tester plates contain histidine deficient media TA98 and TA100 colonies normally cannot develop. Those bacterial colonies that do survive can only do so through reverse mutation, thus indicating probable mutagenesis. Both tester strains were kindly provided by Professor BN Ames, University of California at Berkeley, California, USA through the National Cancer Institute Thailand.

The testing samples generate mutagenicity by inducing reverse mutation in the tester strains during plate incorporation assay according to their mutagenic potential. The number of revertant colonies was counted. All cultures were made in triplicate. The absence of toxicity was examined by observing background bacterial growth, which would normally be present. The positive controls were 4-nitroquinoline 1-oxide (NQO) at a concentration of 21.04 μmol/L for TA98, and 10.52 μmol/L for TA100, Aflatoxin B1 (AFB_1_) at a concentration of 0.96 μmol/L for TA98 and TA100, and benzo(a)pyrene (B(a)P) at a concentration of 396.33 μmol/L for TA98 and 198.16 μmol/L for TA100. The negative controls were distilled water and Dimethyl sulfoxide (DMSO), as they represented the solvents to remove different substance groups in pet foods.

The mutagenicity of the test specimens was assessed by looking at the increase in His + revertants, to at least 20–50 revertant colonies/plate for TA98, and 120–200 revertant colonies/plate for TA100. A test sample produced a positive dose–response relationship over 3 concentrations, with the highest increase not less than twice the negative control value. The trace of histidine on the surface of the agar allowed all the bacteria on the plate to undergo several divisions and produce a faint background lawn, which could be seen under a dissecting microscope. The background lawn is essential to the test as an indicator of growth caused by the toxicity of the test sample. If massive cell deaths occur, defined as the killing effect or partial killing effect, the background lawn on the test plate will be sparse compared with the control plate. In this case, more histidine is available to the surviving bacteria, which undergo more cell divisions and may appear as small colonies, which could lead to false positive results.

### Long-term controlled experimental trial

We used rats to test the long-term effects of the food. A dog’s lifespan is relatively long. The lifespans of rats and dogs are 2–4 and 10–16 years, respectively Compartively, the weaning age for a rat is about 3 weeks (6 weeks for a dog). The experiment started at about 9 weeks of age, or an equivalent 16-20 weeks for a dog. The duration of the study (90 days) was equivalent to 12 % and 2.5 % of the mean lifespan for a rat and a dog, respectively [13].

### Housing

The animal-experimentation protocol was approved by the Ethics Committee of the Faculty of Tropical Medicine-Animal Care and Use Committee. Male Wistar rats with 150–200 g body weight were supplied by the National Laboratory Animal Center, Mahidol University, Salaya Campus, Nakhon Pathom, Thailand. The rats were housed individually in cages in a temperature-controlled room at 25 ± 2 °C with a 12-h light–dark cycle (light on at 6:00 am). All animals were allowed to acclimatize for 2 weeks in the Laboratory Animal Unit of the Faculty of Tropical Medicine, Mahidol University, where they were fed with standard commercial food and distilled water *ad libitum*. Prior to oral subchronic toxicity testing, they were fasted for 16 h and allowed free access to water. The average body weight of the rats at day 0 of the experiment was 310.6 g.

### Data collection

Blood samples were drawn by tail-vein puncture or section every 4 weeks. Hematological analyses of 12 basic parameters were performed by Hemavet (Drew Scientific, Inc.). Blood chemistry was analyzed by the Liasys Analyzer Medical System (AMS) (Rome, Italy), including blood urea nitrogen (BUN), creatinine (CR), aspartate aminotransferase (AST), alanine aminotransferase (ALT), cholesterol (CHOL), triglyceride (TG), and one mineral, phosphorus (P). At the end of the test, after final blood sampling, the rats were euthanized with 10 mg/kg xylazine and 50–70 mg/kg ketamine i.p. Histopathological analysis was in additionally carried out on the liver and kidneys as well as other vital organs such as heart, lung and brain and recorded as lesions under a light microscope after processing in buffered formalin fixation, paraffin embedding, and hematoxylin and eosin stain. The microscopic appearances were recorded.

### Statistical analysis

ONE-WAY ANOVA followed by a Least Significant Different (LSD) and Chi-square test using SPSS version 11.0 for Windows were used to analyze resultants.

## Results

The Ames test findings for the water-extracted diets are shown in Table 1. Mutagenicity was found in all diets under differing conditions. The DG diet was dose-dependent for TA100 in the absence of S9, at a 20 mg/plate and higher concentrations. The AP, AL, and NT diets were positive to TA98, with AP found in the presence of the S9 mix, 20 and 30 mg/plate, but not in the AL diet in the absence of the S9 mix, 20 mg/plate. The NT diet was positive in the absence of the S9 mix at 10–20 mg/plate, and with the S9 mix at 10 mg/plate. The RS diet extract was positive in both tester strains to TA98 without the S9 mix at 5, 10, and 20 mg/plate, and with the S9 mix at 20 and 40 mg/plate, as well as TA100 at 30 mg/plate with the S9 mix. All diets showed partial killing in both tester strains at different concentrations. Diets DG, AP, and NT also exhibited killing effects.

Table 2 shows that the dry-food ethanol extracts were more toxic to the tester bacteria at high concentrations. A partial killing effect with both strains was observed in the absence of the S9 mix at concentrations of 5 and 10 mg/plate, and the presence of S9 mix at 7.5 and 10 mg/plate with the DG diet extract, but no mutagenicity was found. In the AP, AL, RS, and NT diets, dose-dependent mutagenicity was found in different concentrations and S9-mix tests. Mutagenicity was observed in the AP diet extract for TA98 in the absence of the S9 mix, at 1 mg/plate. The AL diet extract produced with TA98 in the absence of the S9 mix, at 0.01 and 0.1 mg/plate. The RS diet produced with TA100 in the absence of S9 mix, at 0.5 and 1 mg/plate. The NT diet produced with TA98 in the presence of S9, at 0.5 mg/plate. Partial killing was observed in all diets at different concentrations. Moreover, killing effects were found in the AP-diet extracts.

**Table 2:** Mutagenicity of dry canine-food extracts from distilled water with *S. typhimurium* TA98 and TA100

Sample	Amount mg/plate	No. of His^+^ Revertants/plate^a^			
		TA 98, -S9	TA 98, +S9	TA100, -S9	TA 100, +S9
DG diet	0	29.5	34	110	123
	5	42.5	49	137.5	133.5
	10	32	55.5	181.5	173
	20	39^p^	68^p^	258.5	190.5^e^
	30	58^p^	70^p^	262.5^p^	235^p^
	40	64.5^p^	87.5^p^	283.5^p^	190^p^
AP diet	0	38	32.5	112	129
	0.5	47	55.5	100	100
	5	48.5	57	129	163
	10	31.5	58	140.5	161.5
	20	39	69	190.5	191.5
	30	42^p^	70.5	191.5^p^	205^e^
	40	102^p^	80.5^p^	207^p^	222^p^
AL diet	0	26.5	34	110	123
	5	43	34.5	125.5	129
	10	41	57	135.5	140
	20	58^e^	58.5^p^	167.5	175
	30	38^p^	63^p^	195^p^	261^p^
	40	37.5^p^	43^p^	189^p^	237.5^p^
RS diet	0	26.5	42	112	129
	0.5	37	42	121	142.5
	5	62	55	150.5	183.5
	10	56	76.5	166	177.5
	20	58.5	80.5	203.5^p^	212
	30	43^p^	67.5^e^	229.5^p^	268^e^
	40	61.5^p^	99.5^e^	261.5^p^	297^p^
NT diet	0	29.5	34	110	123
	5	30.5	57	117.5	171
	10	84	72	162.5	177
	20	68.5	67.5^p^	222.5^p^	224^p^
	30	56^e^	52^p^	236.5^p^	171.5^p^
Positive control	Amount/plate				
4-NQO	0.2 μg	ND	ND	1040±113	ND
	0.4 μg	195±1	ND	ND	ND
BAP	5 μg	ND	ND	ND	887±91
	10 μg	ND	523±4	ND	ND
AFB1	0.03 μg	ND	138.5±3	ND	387.5±3

^a^ **=**Mean of duplicate

^e^=effect

^p^=partial killing effect

ND=Not determine

All rats in both study groups survived the 15-week feeding study. During the experimental period, no significant abnormality in food intake, feces, hair, or behavior in any group of animals was observed, with no apparent toxicity. At the start of the investigation, no significant difference was detected between the rats fed with canine food and the control in all hematological indices, except for mean corpuscular volume.

**Fig. 1:**
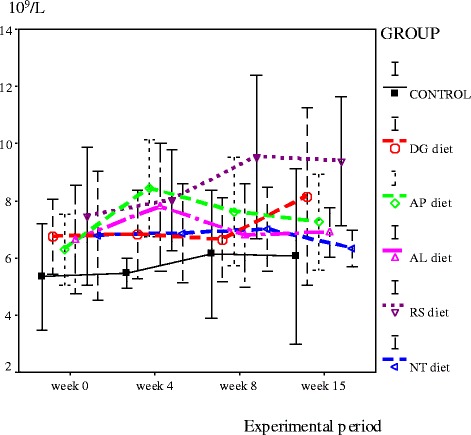
White blood cell count of rats fed experimental diets from week 0 to week 15 (Normal range between 7.3–12.6 ×10^9^/L)

**Fig. 2:**
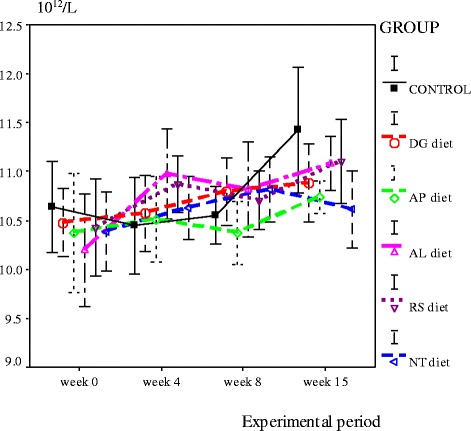
Red blood cell count of rats fed experimental diets from week 0 to week 15 (Normal range between 6.6–9.0 ×10^12^/L)

At week 4 of the experimental period, blood parameters in rats fed with 3 diets AP, AL and RS diets) were more elevated, some statistically significantly. These included red blood cells (RBC), hemoglobin (Hb), hematocrit (Hct), mean corpuscular hemoglobin (MCH), mean corpuscular hemoglobin concentration (MCHC), platelets, white blood cells (WBC), and monocytes (*P* < 0.05). At weeks 8 and 15, most hematological indices of the control group increased sharply, but the level was still not significantly different from the rats fed with canine food. Interestingly, the white blood cell counts of the control group of rats were lower than all dry-canine-food groups for all 15 weeks of the experiment. Thus, food quality may affect WBC numbers. In addition, the RBC count for all dry-canine-food groups, and the control group, were higher than the normal range. Thus, blood-sampling site may affect blood concentrations and RBC numbers (Figs. 1 and 2).

In the long-term feeding experiment with dry canine foods, serum cholesterol and TG levels of the experimental groups were slightly higher than the control group, but without statistical significance. Among the rats fed dry canine foods, serum blood chemistry results (BUN, CR, ALT, AST, and phosphorus) were statistically different from the control group. However, these blood parameters were normal, except that BUN was higher than the control rats. Gross examinations of the organs (heart, liver, kidneys, spleen, pancreas, and other internal organs) were conducted at week 15, and no changes in any rat were noted. Histopathologic changes were blindly examined by two independent observers (UC and PP). The liver and kidneys were graded into 5 levels; normal appearance, 0–25 %, 26–51 %, 51–75 % and 76–100 % tissue degeneration.

**Fig. 3:**
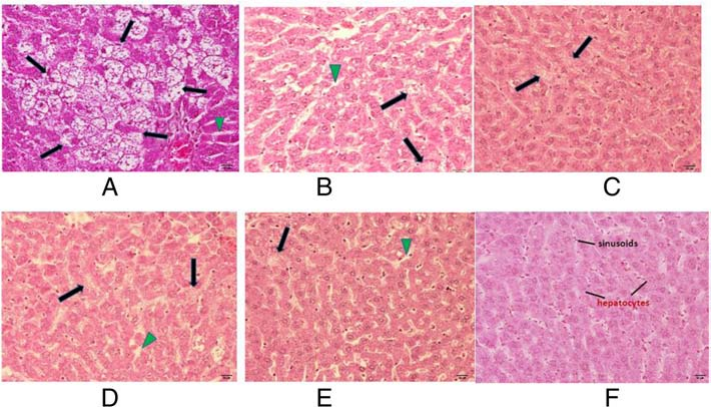
Paraffin section of male rat livers with hematoxylin and eosin staining at x 40 magnification. The rats were fed with DG, AP, AL, RS and NT canine diet for 90 days. Arrows were indicating abnormal liver appearance of rats with foamy cells. Arrow heads were indicating abnormal liver appearance of rats with the dilation of sinosoids

As seen in Table 3 and 4, the liver and kidney of all rats fed with dog foods were significantly degenerated (*p* < 0.05) compared to the control group. In agreement with the higher amount of ALT and AST, liver histopathology of all dry-canine-food groups showed irregular phenomena when compared to the control group. Under microscopic examination at 40 times magnification, the livers of control rats were seen to be composed of many individual polygonal hepatocytes with a well defined plasma membrane. There was a normal appearance of space between hepatocyte sinusoids containing a few red blood cells. In contrast, the livers of rats treated with dry canine foods presented marked degeneration of the tissue, especially in the DG diet (Fig. 3). These showed nonspecific areas of hepatocytes with scattered shapes and sizes. The abundant foamy cells (arrow) indicate the fat deposition. The dilation of sinosoids (head arrow) which usually accompanies hypertrophy and hyperplasia, which all caused by tissue injury was also observed.

**Table 3:** Mutagenicity of dry canine-food extracts from absolute ethanol with *S. typhimurium* TA98and TA100

sample	Amount mg/plate	No. of His^+^ Revertants/plate^a^			
		TA 98, -S9	TA 98, +S9	TA100, -S9	TA 100, +S9
DG diet	0	32	30	122	110
	0.1	30	50	91	86.5
	0.5	33	41	129	92
	1	36.5	52	114	90.5
	5	19^p^	44	85^p^	84.5
	7.5	ND	47^e^	ND	90.5^e^
	10	13.5^p^	11.5^p^	ND	76.5^p^
AP diet	0	28	27	137	110
	0.01	26.5	ND	116	ND
	0.1	25	39	116.5	87
	0.5	29.5	38.5	84.5	90.5
	1	55	33	61^p^	87
	5	25.5^p^	33.5	76.5^p^	81
	10	26^p^	45.5^p^	100.5^p^	103.5^e^
	20	31.5^p^	30^p^	86^p^	100^e^
	30	21^p^	ND	105.5^p^	ND
AL diet	0	28	30	137	110
	0.01	66	ND	113	ND
	0.1	63	38.5	94.5	91
	0.5	64.5^p^	49	83^p^	103
	1	32^p^	56.5	94.5^p^	107.5
	5	ND	31.5^p^	ND	132.5
	10	ND	21^p^	ND	ND
RS diet	0	32	26	137	110
	0.01	27	ND	ND	ND
	0.1	48.5	33	196.5	92
	0.5	31^p^	42.5	307.5	126
	1	37.5^p^	30	262.5	138.5
	7.5	ND	28.5	63^p^	87.5^p^
	10	ND	ND	75^p^	106.5^p^
NT diet	0	32	30	108	110
	0.1	31.5	34.5	69.5	101.5
	0.5	28.5	63	78^p^	84.5
	1	31	52.5	95.5^p^	127.5
	2.5	13.5	33	ND	126
	5	21^e^	81^p^	ND	109^e^
	10	13.5^p^	58^p^	ND	ND
Positive control	Amount/plate				
4-NQO	0.2 μg	ND	ND	ND	ND
	0.4 μg	227.5±3	ND	ND	ND
BAP	5 μg	ND	ND	1430±14	863±75
	10 μg	ND	459±12	ND	ND
AFB1	0.03 μg	ND	130±21	ND	241±12

^a^ **=**Mean of duplicate

^e^=effect

^p^=partial killing effect

ND = Not determined

**Table 4:** Summary of histopathological appearance in kidney and liver of rats

*Organ appearance*	DG diet		AP diet		AL diet		RS diet		NT diet		CONTROL
	n	p	n	p	n	p	n	p	n	p	n
Kidney											
normal appearance	-	0.007	-	0.007	-	0.001	-	0.007	-	0.017	6
0–25 % tissue degenerated	2		1		6		2		1		-
26–50 % tissue degenerated	2		2		-		2		3		-
51–75 % tissue degenerated	2		3		-		2		1		-
76–100 % tissue degenerated	-		-		-		-		1		-
Total	6		6		6		6		6		6
Liver											
normal appearance	-	0.007	-	0.017	-	0.002	-	0.007	-	0.002	6
0–25 % tissue degenerated	2		2		4		-		5		-
26–50 % tissue degenerated	1		1		2		1		1		-
51–75 % tissue degenerated	-		2		-		4		-		-
76–100 % tissue degenerated	3		1		-		1		-		-
Total	6		6		6		6		6		6

The numbers of rats were 6 per each group. Values are significantly different at *P* < 0.05 using Chi-Square test compared to control

**Fig. 4:**
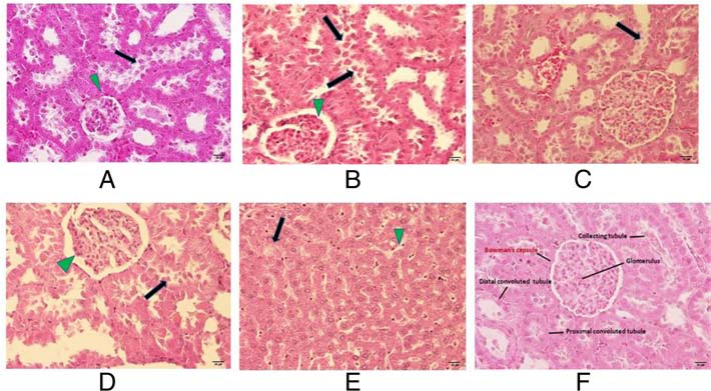
Paraffin section of male rat kidneys with hematoxylin and eosin staining at x40 magnification. The rats were fed with DG, AP, AL, RS and NT canine diet for 90 days. Arrows were indicating kidney degeneration of rats with the collapsed epithelial cells of the proximal tubules. Arrow heads were indicating kidney degeneration of rats with the congested and atrophied glomerulus

Under light microscopy at 40 times magnification, the illustration of kidney histopathology is also in agreement with the BUN results. The cortical area of the control group displayed a regular round structure of the glomerulus with the continuous layer of Bowman’s capsule. The cells of the collecting tubules, proximal convoluted tubules and distal convoluted tubules were intact with distinct cell outlines. However, higher amounts of BUN were seen in all dry-canine-food groups along with the same kidney degeneration, especially in the NT diet (Fig. 4). The epithelial cells of the proximal tubules had degenerated and collapsed into the lumen (arrows). The glomerulus was also found to be congested and atrophied (arrow head), resulting in the loss of reabsorption and filtration functions. No pathological changes appeared in heart, lung and brain.

## Discussion

Short-term tests that detect genetic damage have allowed the carcinogenic risk of chemicals to humans to be evaluated [14]. The Ames assay, which is recommended for testing the mutagenicity of chemical compounds with potential pharmacological applications [15, 16] was used in the present study. The mutagenic test used *S. typhimurium* tester strains TA98 and TA100, from two kinds of solvent extracts (distilled water and absolute ethanol). They showed significantly different numbers of induced revertants compared with the control group.

Four of five samples of local-brand dry canine foods extracted by distilled water showed a considerable mutagenic effect on the TA98 strains, which detect frameshift-type mutagens, while two of the samples yielded positive with TA100, a strain that reverts by means of base-pair substitution mutagens. In addition, three samples extracted by absolute ethanol had a considerable mutagenic effect on TA98. Thus, the types of response in different strains clearly differed, revealing significant levels of frameshift-type mutagens in the samples. Frameshift mutations are likely to result in more severe phenotypic effects than many base-change mutations, which result in either silent or conservative changes in protein products [17]. The positive results in our study could be attributed to the extracting agent and the fat content of the pet foods, which affect the properties of the extractor.

Pet-food-extract samples showed mutagenic activity up to a concentration of 20 mg/plate; the highest water-extraction concentration was 40 mg/plate. Water extracts of some dry canine foods at the tested concentrations (up to 50 mg/plate) showed toxicity to the tester strains, with or without a metabolic activator (S9 mix). Some ethanol-extracted dry canine food, with or without the S9 mix, an antimicrobial effect (toxicity) toward tester strains was also detected when the concentration was >10 mg/plate. According to the results of the toxicity test, the highest concentrations of dry canine food extracts for the mutagenicity assay were selected as 40 mg/plate for water extracts, and 10 mg/plate for absolute ethanol extracts. On the other hand, absolute ethanol extracts exhibited strong antimicrobial activity toward *S. typhimurium;* this result led us to suppose that ethanol was as stronger solvent, and more mutagenic agents could be extracted. To confirm the potential effects of the diets, further in vivo studies are needed using animal models, to isolate the components of dry canine food.

Knize et al. [18] analyzed twenty-five commercial pet foods for mutagenicity activity using the Ames’/Salmonella test with strain TA98 and added metabolic activation. Almost all gave a positive mutagenic response [18]. This may be affected from various substances contained. Dry pet food contains a number of nutrients and additive compounds. The diets also contain cooked meat. Differences might be expected by the type of meat, heating process. On the other hand, several substances related to natural antimutagenicity or anti-carcinogenicity that has simultaneously been identified as mutagenic or carcinogenic [19]. The positive findings in this study revealed the presence of mutagenic components in dry pet-food extracts, with either direct or indirect mutagenic action. Future studies should focus on better characterization of the mutagenic and antimutagenic activities of dry pet food extracts, by using other tester strains, and identifying specifically their active compounds and modes of action.

Although no significant effect of long-term consumption of dry canine foods (for 15 weeks) in rats was observed on clinical blood chemistry, total average weight gain in some groups of rats fed commercial canine foods was higher than the control. There were also differences in clinical blood chemistry for serum BUN, CR, ALT, AST, and P, compared with the control group. This may have resulted from the different grades of dry canine foods. These are in agreement with our histopathological analysis, which showed that all 5 brands of dry canine foods induced the pathophysiological degeneration of the liver and kidney. Overconsumption of pet food may cause the change in gut bacteria. Although the underlying mechanism is not yet known, changes in microbial diversity in the intestine may potentially contribute to fat accumulation and degeneration of the liver and kidney. Some photochemicals such as ursolic acid have been reported to change the amount of gut bacteria and decrease the risk of obesity [11]. The composition of fatty acids in pet food may influence the expression of amino acids in the liver and kidney [10]. This may also be the cause of degeneration of liver and kidney. In addition to over consumption of foods, several studies reported that most canine food was contaminated with aflatoxin [20], some trace metal elements [21] and heterocyclic amine from heating process during food production [18]. All these can cause liver and kidney degeneration, due to increased rates of tissue oxidative stress and toxic chemical production [20, 21].

All rats remained alive at the end of the experimental period (15 weeks) without any externally apparent signs of toxicity. However, there were mild abnormal blood biochemistry results, and histological changes in canine food fed rats after sacrifice. No change in food intake was observed in any groups. Body weight and body weight gain appeared to not be affected. A further study is planned to investigate the toxic effects of local dry canine foods following OECD guide lines, in which acute and chronic effects analysis with varying doses and time. This would include investgating ultrastructural changes to find early degeneration in vital organs. Moreover, a cohort study with dogs could provide further explanation of the dynamics over time.

## Conclusions

Mutagenic potential was found in all local-brand dry canine foods tested, and the potential health impacts of long-term consumption in rats were observed. Thus, the dog owner should be aware of their commercial dog food choices or alternatively feed their dog with fresh food. Further mutagenicity testing and identification of toxic substance contamination in the diet should be conducted, to elucidate the safety of locally manufactured, generic-brand, dry canine foods.

## Abbreviations

AFB_1_: Aflatoxin B_1_
AFB_2a_: Aflatoxin B_2a_
ALT: Alanine aminotransferase
AMS: Analyzer Medical System
AST: Aspartate aminotransferase
B(a)P: Benzo(a)pyrene
BUN: Blood urea nitrogen
CHOL: Cholesterol
CR: Creatinine
Hb: Hemoglobin
Hct: Hematocrit
LSD: Least Significant Different
MCH: Mean corpuscular hemoglobin
MCHC: Mean corpuscular hemoglobin concentration
NQO: 4-Nitroquinoline 1-oxide
P: Phosphorus
TG: Triglyceride
US: United States
WBC: White blood cells
